# Epstein-Barr virus-positive iris diffuse large B-cell lymphoma detected by metagenomic next-generation sequencing

**DOI:** 10.1186/s12886-024-03334-8

**Published:** 2024-03-04

**Authors:** Xiao-na Wang, Jing Hong, Yong-gen Xu, Pei Zhang, Ying-yu Li, Hong-liang Dou, Hai-ping Li

**Affiliations:** Department of Ophthalmology, Beijing Key Laboratory of Restoration of Damage Ocular Nerve, Peking University Third Hospital, 100191 Beijing, China

**Keywords:** Epstein-Barr virus, Lymphoma, mNGS

## Abstract

**Purpose:**

Epstein-Barr virus (EBV)-positive diffuse large B-cell lymphoma (DLBCL) is a relatively rare subtype of DLBCL. Herein, we report a case of a patient with EBV-positive iris DLBCL after undergoing penetrating keratoplasty and discuss its possible pathogenesis.

**Methods:**

A 72-year-old male patient presented to our hospital with progressive blurring of vision in the left eye for the past 4 months. Small white nodular lesions were observed on the iris and retinal surface of the left eye, with a white cloud-like opacity in the vitreous cavity.

**Results:**

The patient was eventually diagnosed with EBV-positive iris DLBCL after undergoing pathological and metagenomic tests. After injecting methotrexate in the left vitreous cavity and administering systemic and local antiviral treatments, the ocular lesions disappeared.

**Conclusion:**

EBV infection, drug immunosuppression, and aging-related immune deterioration may play significant roles in the pathogenesis of EBV-positive iris DLBCL.

**Synopsis:**

Epstein-Barr virus (EBV)-positive diffuse large B-cell lymphoma (DLBCL) is a new subtype of DLBCL, which rarely occurs. Herein, we report a case of a patient with EBV-positive iris DLBCL after undergoing penetrating keratoplasty and discuss its possible pathogenesis.

## Introduction

Intraocular lymphoma is a rare type of ocular malignancy and has two types: primary and secondary. Primary intraocular lymphoma (PIOL), a subset of primary central system lymphoma (PCNSL), affects the vitreous, retina, iris, ciliary body, choroid, or optic nerve, without systemic lymphoma (PCNSL). Most patients diagnosed with PIOL have diffuse large B-cell lymphoma (DLBCL) [1]. However, diagnosing PIOL remains challenging. Differential diagnoses should be made by identifying the infectious and non-infectious etiologies, particularly common masqueraders such as sarcoidosis, tuberculosis, and viral retinitis. Epstein-Barr virus (EBV)-positive DLBCL in older adults is a new subtype of DLBCL according to the 2008 World Health Organization classification. However, this subtype is extremely rare. Herein, we report a case of a patient with EBV-positive iris DLBCL after undergoing penetrating keratoplasty.

### Case presentation

A 72-year-old male patient presented to our hospital due to progressive blurring of vision in the left eye for the past 4 months. Thirty-two years ago, the patient had undergone left eye cataract phacoemulsification without intraocular lens implantation due to high myopia. Eight months ago, the patient was diagnosed with corneal endothelial decompensation, corneal leukoplakia, and aphakia of the left eye after undergoing examination in our eye center. Penetrating keratoplasty, intraocular lens implantation in the ciliary groove by suturing, and anterior vitrectomy of the left eye were performed. Corticosteroid eye drops and anti-immunological rejection therapy were administered as maintenance therapy for 6 months postoperatively. The patient’s visual acuity substantially improved. However, 4 months later, the patient presented with blurred vision and redness in the left eye.

**Fig. 1:**
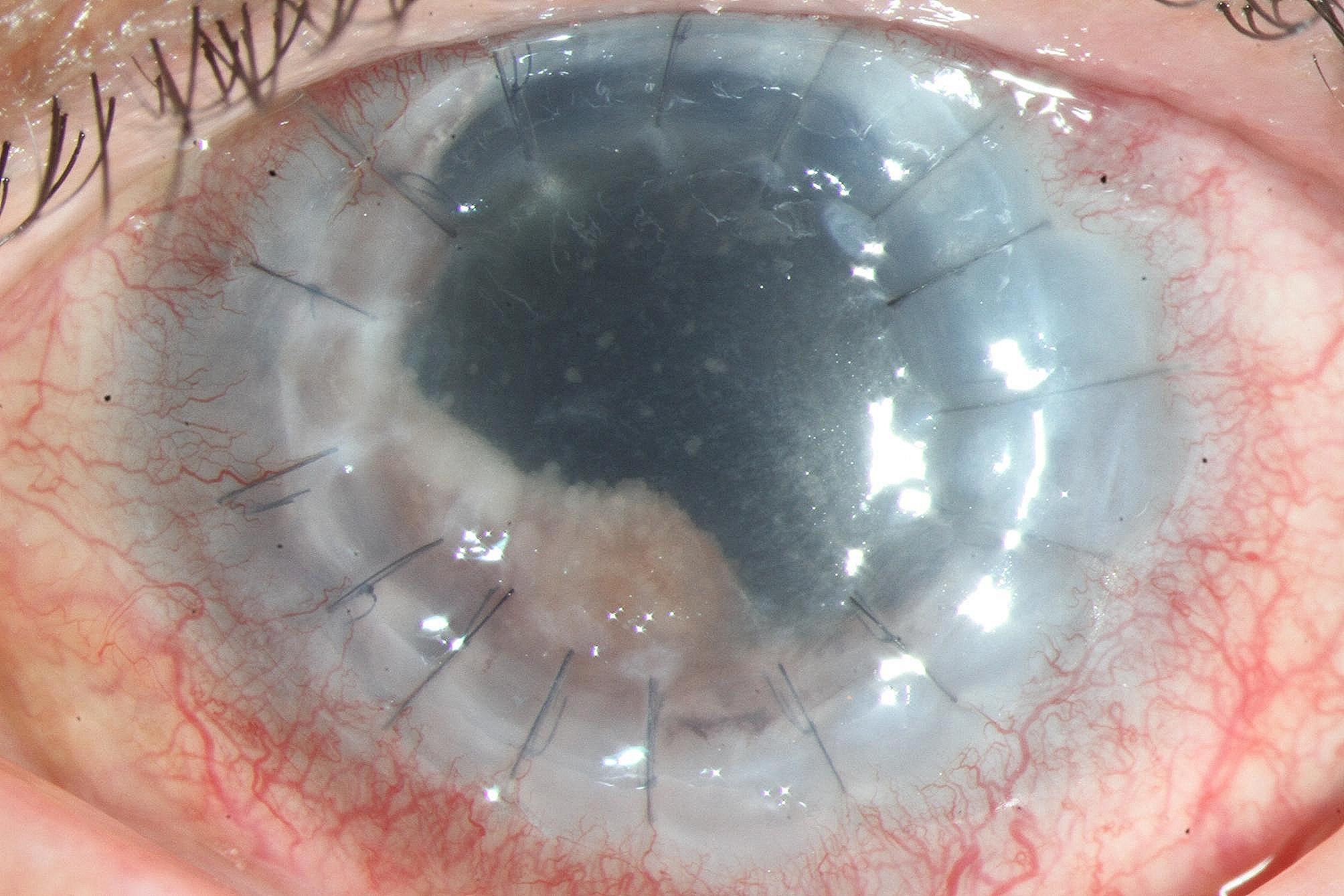
Anterior segment photo shows graft cornea display transparency and residual iris with thick, pink, and whitish small nodules on the surface

Physical examination: The left eye was only sensitive to hand movements and had an intraocular pressure of 18 mmHg. The left eye presented with conjunctival hyperemia, mild edema of the grafted cornea, 3 + cells and fibrin in the anterior chamber, and superior and temporal iris defects (Fig. 1). Small white nodule lesions were noted on the iris and retinal surface, while a white cloud-like opacity was observed in the vitreous cavity. Infectious endophthalmitis, especially fungal infections, was strongly suspected.

**Fig. 2:**
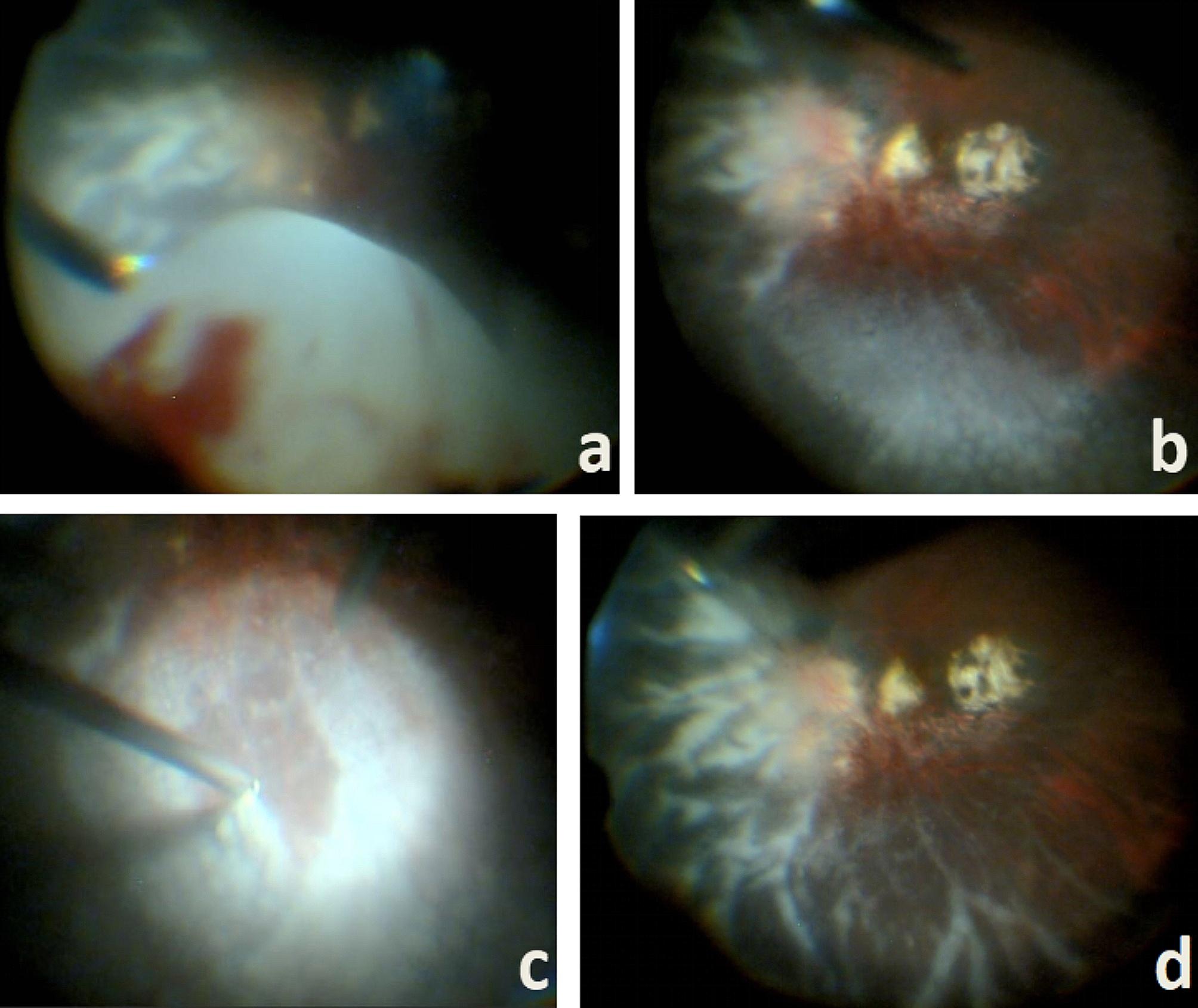
Vitreous and retina images during surgery: (**A**) white cloudy vitreous opacity, (**B**) white dense nodules on the inferior retinal surface, (**C**) section of white dense nodules, and (**D**) white sheath-like exudates can be observed after the removal of white nodules from the retinal surface

Vitrectomy was performed on the left eye owing to the probable diagnosis of infectious endophthalmitis. During surgery, retinal vascular sheath and retinal hemorrhage spots were observed. White cloudy vitreous opacities and diffuse nodule lesions arranged in a carpet-like appearance on the retinal surface were noted. Patchy atrophy of the choroid and retina was also detected at the posterior pole due to myopia (Fig. 2). Part of the iris tissue was removed. Hence, aqueous humor and undiluted vitreous humor samples were collected to examine the presence of bacteria, viruses, cells, and other pathological agents.

Aqueous humor virus polymerase chain reaction (PCR) test showed an EBV deoxyribonucleic acid (DNA) level of 3.82 × 10^7^ copies/ml. The interleukin (IL)-6 and IL-10 levels in the aqueous humor were 7.7 × 10^4^ pg/ml and 171.29 pg/ml, respectively. Microbial cultures of vitreous samples were negative for bacteria and fungi. The final pathological test showed an iris EBV-positive DLBCL. Immunohistochemical tests revealed CD20(+), CD3(−), Muml(+), CD10(−), Bcl-6(−), Melan A(−), S-100(−), HMB45(−), EMA(−), CD68(−), Ki-67 (70%+), BCL2(>90%+), c-Myc(40%+), CD5(−), Cyclin D1(−), CD19(+), CD30(70%+), and EBV-EBER(80%+). The fluorescence in situ hybridization assay showed absence of c-Myc.

**Fig. 3:**
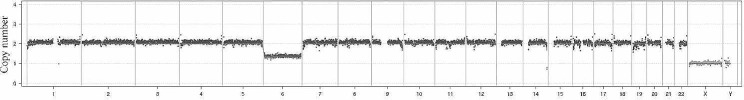
CNV analysis of the vitreous sample from this patient shows an obvious loss of chromosome 6

Considering the possibility of a tumor arising from the left eye, copy number variation analysis was performed using homo-reads in the sequencing data. Unexpectedly, the vitreous sample obtained from this patient showed an obvious loss of chromosome 6. This finding suggests that the patient may have had cancer (Fig. 3).

To further explore other possible pathogens, metagenomic next-generation sequencing (mNGS) was performed. Briefly, 50 uL of vitreous body was used for nucleic acid extraction, followed by fragmentation, adapter ligation, and library purification. The purified library was loaded in the NextSeq 550Dx system to perform sequencing. After removing the adapter self-ligation and filtering low-quality reads, 16.6 million reads were obtained. These data were then entered into a standardized metagenomic analysis pipeline. A total of 8, 307 EB reads were obtained with a relative abundance of 95.59%. Other microbes common in the environment, such as *Pseudomonas fluorescens* and *Acinetobacter johnsonii*, were detected in small amounts.

After diagnosing iris EBV + DLBCL through pathological and immunohistochemical examinations, systemic positron emission tomography/computed tomography (PET/CT) was performed. Postoperative changes in the left eye were identified on PET/CT, with a maximum standardized uptake value of 7.1. No abnormalities were noted in other organs. Results of routine blood tests, liver and kidney assays, and coagulation function tests were normal. Particle agglutination test detected the presence of *Treponema pallidum*. Viral PCR testing of corneal transplant donors was also performed, which yielded negative results for herpes simplex virus I, II, cytomegalovirus, varicella-zoster virus, and EBV.

The patient was treated with oral valacyclovir for 3 months. Levofloxacin, ganciclovir gel, tacrolimus, and loteprednol ophthalmic suspensions were administered for 3 weeks. Following the diagnosis of DLBCL by pathological, immunohistochemical, and metagenomic tests, 400 micrograms/0.1 ml of methotrexate (MTX) was injected into the vitreous cavity of the left eye twice every two weeks. After receiving two intravitreal injections, the patient refused to continue the MTX treatment.

**Fig. 4:**
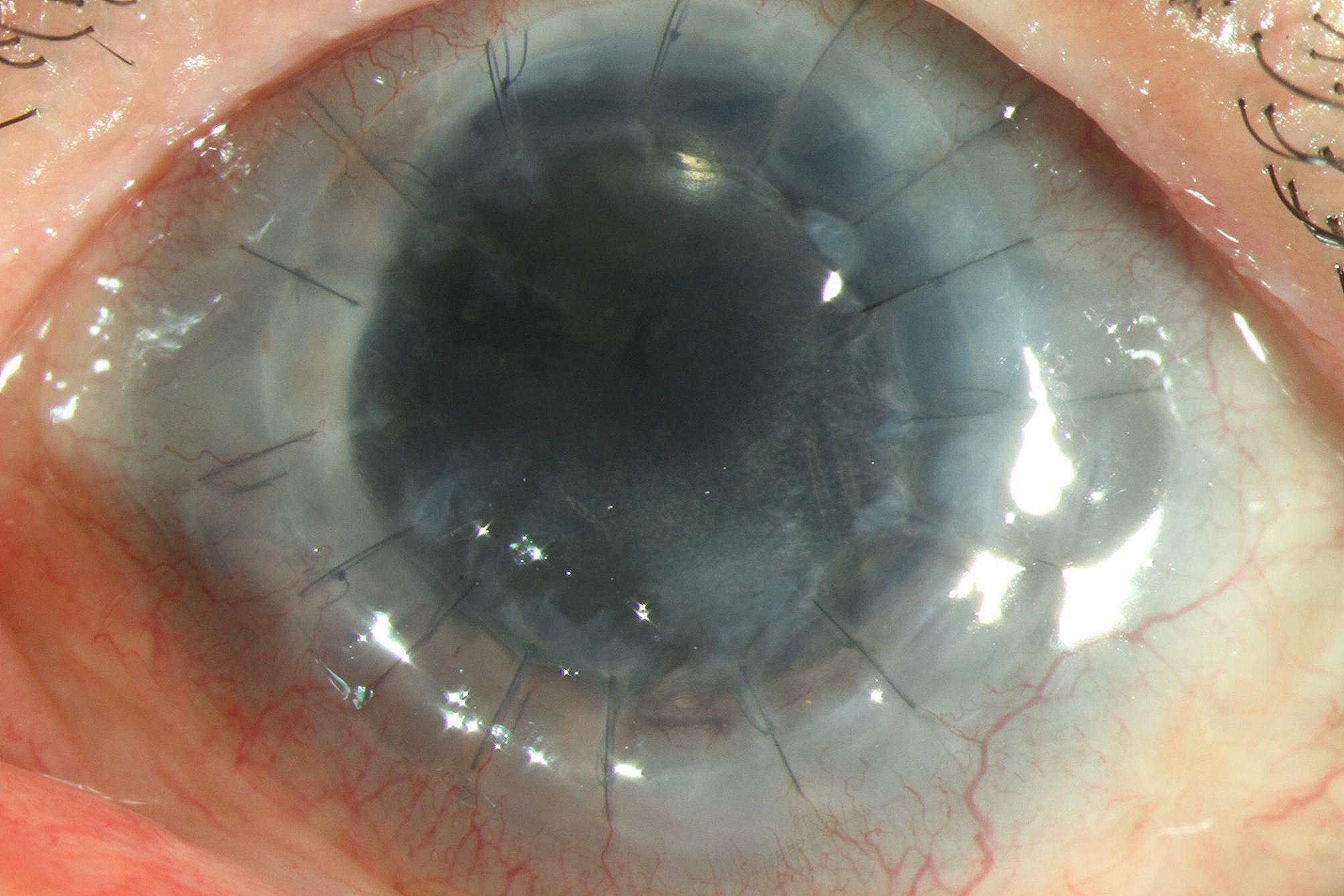
Graft cornea image of a graft cornea with approximate transparency

**Fig. 5:**
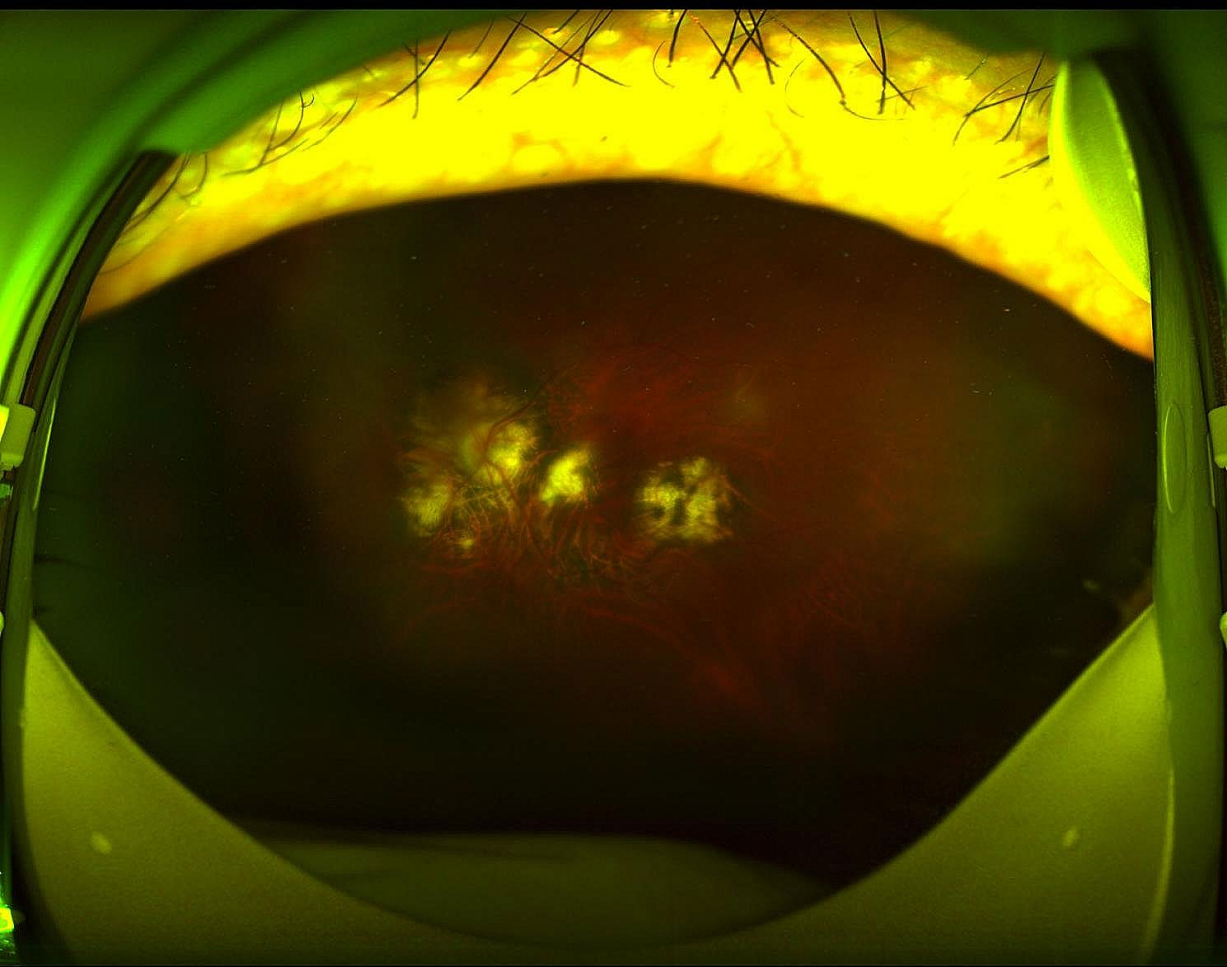
The white nodules on the retinal surface and retinal vessel white sheath completely disappeared; the choroid and retina at the posterior pole appeared patchy atrophy within 4 months after undergoing vitrectomy and after receiving antiviral treatment and two intravitreal injections of MTX

Four months later, the BCVA of the left eye increased to 20/400, but the intraocular pressure remained normal. The graft cornea displayed approximate transparency, the retinal vasculitis subsided, and the white surface nodules were no longer detectable on the colorful fundus picture (Figs. 4 and 5).

The content of this article was submitted after the patient’s verification, and an informed consent form was signed. The study design was approved by Peking University Third Hospital Medical Science Research Ethics Commitee.

## Discussion

Ocular EBV infections have various clinical manifestations, including scleritis, posterior uveitis, retinal vasculopathy, retinitis, acute retinal necrosis, and intraocular lymphoma [2]. Although rare, the incidence of intraocular lymphoma has increased in recent years, but its etiology remains unknown. Herein, we report a case of a patient with EBV-positive DLBCL in the iris after undergoing penetrating keratoplasty. Through analysis of this special case, we were able to identify the cause of intraocular lymphoma.

First, the incidence of EBV-induced lymphoid or epithelial malignancies is well recognized [3]. Transmission of the virus occurs mainly through exposure to oral secretions, but EBV infection through organ transplantation has also been reported. The two main physiological targets of EBV are the epithelial cells and B lymphocytes. EBV replication occurs primarily in the epithelial cells and establishes a latent infection in the B lymphocytes, where it expresses limited sets of proteins called EBV transcription programs to efficiently transform B cells into lymphoblastic cells. In immunocompetent individuals, the levels of viral titers are determined by measuring the amount of EBV-specific cytotoxic T cells [4]. In normal histological structures, the iris possesses several epithelial cells and lymphocytes; therefore, it is prone to developing EBV-related lymphoma.

PIOL is considered to be a part of PCNSL. 15–20% of all PCNSL patients have PIOL at presentation; 50–90% of patients with PIOL develop a CNS and/or spinal cord disease after 16–24 months [5]. In 2011, the International PCNSL Collaborative Group published therapeutic principles for VRL. According to the group, systemic therapy is required if lymphoma lesions occur in the CNS and local therapy may be used if the disease is limited to the eye [6]. There is currently no standardized treatment plan for Epstein-Barr virus positive iris lymphoma in the literature [7, 8]. Because the lesion in our patient was relatively limited without systemic metastasis, the visual acuity of the left eye improved, and the white dense opacity in the vitreous cavity and retinal surface nodules completely disappeared after performing vitrectomy combined with long-term antiviral therapy and two intravitreal injections of MTX. In the subsequent treatment process, the patient was unwilling to cooperate with the examination and treatment due to personal reasons, and ultimately lost follow-up.

In addition, the patient received long-term ocular immunosuppressive therapy, including steroids and tacrolimus eye drops, after undergoing penetrating keratoplasty. Steroid eye drops can compromise the ocular immunity. Additionally, tacrolimus eye drops comprehensively inhibit the T-cell function in the eye. Collectively, these factors predisposed our patient to EBV infection in the ocular region.

Aside from ocular immune depression, aging-related systemic immunological deterioration is also one of the causes of EBV-positive DLBCL. The median age of patients with EBV-positive DLBCL was 71 years (range: 50–91 years) [9]. Age-related decline in cellular immunity is caused by several factors, including thymic atrophy, reduced output of new T lymphocytes, accumulation of anergic memory cells, deficiency in cytokine production, and uncertain antigen presentation [9]. Therefore, a decline in age-related immune function may also play a role in the occurrence and development of this disease.

To our knowledge, this case report is the first to document a case of a patient with an EBV-positive iris DLBCL; the diagnosis was established not only through pathological and immunohistochemical analyses, but also through metagenomic tests. In recent years, with the emerging of pathogen detection technology, mNGS has been increasingly used for the detection of various infections, such as bloodstream infection, [10] central infection, [11] and respiratory infection [12]. In eye infections, metagenomics also has some valuable applications, facilitating the prompt initiation of treatment with an optimal antimicrobial regimen [13, 14]. Metagenomic data do not only contain microorganism reads. Human sequences accounted for more than 95% of the mNGS data. Gu et al. [15, 16] reported that the homo reads in mNGS data could be used to identify tumor clues in body fluid and cerebrospinal fluid samples by conducting a copy number variation analysis. Based on the dual-analysis pipeline, mNGS can simultaneously analyze clues of infection and tumors. In this study, we identified a large number of EBV viruses using a metagenomic microbial analysis pipeline. Meanwhile, obvious chromosomal abnormalities, such as monosomy 6, were also identified in this sample, and the pathological results confirmed that the patient had EBV-positive iris DLBCL.

Although the patient’s diagnosis was confirmed, there was a need to differentiate it from other disorders. Post-transplant lymphoproliferative disorder (PTLD) is a malignancy with life-threatening complications occurring in recipients of both solid organ and hematopoietic stem cell allografts [17]. Lymphoma occurs as a result of B-cell proliferation after transplantation and is mostly caused by EBV infection [18]. The use of immunosuppressants leads to the depletion of EBV-specific T cells and disruption of the immune system balance, which eventually causes PTLD in transplant recipients [19]. Chen reported a case of a young patient who developed an iris tumor with mutton-fat keratic precipitates after undergoing a liver transplantation surgery; anterior uveitis and iris nodules were the most common ocular manifestations of PTLDs [20, 21] No study has reported a case of post-keratoplasty lymphoproliferative disorders; the clinical manifestations in our patient were not consistent with those of PTLD. Although the diagnosis of PTLD can be excluded, the negative effects of ocular immunosuppressants on the activation of intraocular viruses and the disruption of intraocular immune balance should be considered.

There are still some limitations in this case report, such as the short follow-up time in patients who have been treated and not been treated regularly in the hematology department. In this case, EBV infection, drug immunosuppression, and age-related immune deterioration may have played a role in the pathogenesis of EBV-positive iris DLBCL. EBV infection and the existence of chromosomal abnormalities in tumor cells were confirmed for the first time through metagenomic sequencing. This is a novel method for the diagnosis of intraocular tumors.

## Data Availability

No datasets were generated or analysed during the current study.
